# Cause of Lamp Explosions

**Published:** 1899-11

**Authors:** 


### Cause of Lamp Explosions.

A curious result has been arrived at in the investigation of the cause of lamp explosions in London, and that is, it is not the worst or lowest-flash oils which are most dangerous, but those of medium grade. For instance, in fifteen explosions eleven were caused by medium oils with a flash-point of from eighty to eighty-eight degrees, and only four by oils of from seventy-three to eighty degrees. At the same time glass lamps are found more dangerous than metal ones, not because they are more liable to break, but because they are cooler; the secret of these apparent paradoxes being that oil vapor is explosive only when mixed with air in the exact proportion of forty-one to fifty, which is quickly exceeded in the hot metal lamp-bowls and with the volatile, low-flash oils.—*The Journal.*

